# The Medicines Optimisation Innovation Centre: a dedicated centre driving innovation in medicines optimisation-impact and sustainability

**DOI:** 10.1007/s11096-024-01775-1

**Published:** 2024-07-23

**Authors:** A. Hogg, M. Scott, G. Fleming, C. Scullin, R. Huey, S. Martin, N. Goodfellow, C. Harrison

**Affiliations:** 1 Medicines Optimisation Innovation Centre, Antrim, Northern Ireland; 2 Department of Health, Belfast, Northern Ireland

**Keywords:** Clinical pharmacy service, Health policy, Pharmacy research, Patient participation, Patient safety, Technological innovations

## Abstract

**Background:**

Sub-optimal medicines use is a challenge globally, contributing to poorer health outcomes, inefficiencies and waste. The Medicines Optimisation Innovation Centre (MOIC) was established in Northern Ireland by the Department of Health (DH) in 2015 to support implementation of the Medicines Optimisation Quality Framework.

**Aim:**

To demonstrate how MOIC informs policy and provides support to commissioners to improve population health and wellbeing.

**Setting:**

MOIC is a regional centre with multidisciplinary and multi-sector clinical expertise across Health and Social Care and patient representation.

**Development:**

Core funded by DH, MOIC has a robust governance structure and oversight programme board. An annual business plan is agreed with DH. Rigorous processes have been developed for project adoption and working collaboratively with industry.

**Implementation:**

MOIC has established partnerships with academia, industry, healthcare and representative organisations across Europe, participating in research and development projects and testing integrated technology solutions. A hosting programme has been established and evaluation and dissemination strategies have been developed.

**Evaluation:**

MOIC has established numerous agreements, partnered in three large EU projects and strengthened networks globally with extensive publications and conference presentations. Informing pathway redesign, sustainability and COVID response, MOIC has also assisted in the development of clinical pharmacy services and antimicrobial stewardship in Europe and Africa. Northern Ireland has been recognised as a 4-star European Active and Healthy Ageing Reference Site and the Integrated Medicines Management model as an example of best practice in Central and Eastern Europe.

**Conclusion:**

MOIC has demonstrated considerable success and sustainability and is applicable to health systems globally.

## Facilitators of best practice

- Department of Health core funding, strategic direction and support and link to informing policy to improve patient care.
- Collaboration with stakeholders across academia, industry, healthcare, policy makers, patients and representative organisations are key to MOIC’s success.
- Partnerships and engagement across Europe facilitate knowledge transfer and opportunities for large European funded projects.

## Barriers to best practice

- UK exit from EU and uncertainty about UK association to Horizon Europe. The UK has now associated and MOIC can continue to participate in European funding calls.
- Workforce and service pressures within healthcare, impacting on the availability of clinical staff for projects and hosting. Work is underway by the Department of Health to develop the pharmacy workforce.
- Covid-19 pandemic as projects were paused. Health and Social Care has now reset and project work has recommenced.

## Background

Sub-optimal medicines use is a challenge globally, contributing to harm, poorer health outcomes, inefficiencies and waste [1, 2]. In healthcare, 1 in 20 patients experience preventable medication-related harm [3]. Sub-optimal use of antimicrobials, contributing to antimicrobial resistance, is one of the top 10 global public health threats and the focus of the European One Health Action Plan [4]. The ageing population and increasing numbers of people living with multiple long term conditions have escalated demand for medicines [5]. Northern Ireland (NI) consumes more medicines, including antimicrobials and analgesics, than other parts of the United Kingdom (UK). The mean number of prescription items per person per year in NI is 23, costing £245, the highest in the UK [6].

The environmental impact of pharmaceuticals, including antimicrobial resistance and loss of biodiversity is a growing concern, requiring urgent action and the introduction of ‘eco-directed and sustainable pharmaceutical prescribing’ [7]. Constrained resources, workforce pressures and the need to rebuild following the Covid-19 pandemic, further compound the unprecedented challenges faced by healthcare systems worldwide. Innovation in medicines optimisation, embracing pathway redesign, behavioural science, sustainability, pharmacogenomics, artificial intelligence and integrated technology solutions, including companion diagnostics, is needed to transform healthcare systems, supporting the delivery of high quality, safe, effective and sustainable person-centred care [8–10].

In 2016, the NI Department of Health (DH) published the NI Medicines Optimisation Quality Framework [11]. The aim was to support better health outcomes for the population through gaining the best possible outcomes from medicines every time they are prescribed, dispensed or administered. The Framework focussed on the consistent delivery of best practice and supported the development and implementation of new, evidence based best practice delivered through an innovation and change programme involving multi-disciplinary professionals working together and with patients.

The Medicines Optimisation Innovation Centre (MOIC) was established by the DH in 2015 to support implementation of the Medicines Optimisation Quality Framework.

### Aim

To demonstrate how MOIC informs policy and provides support to commissioners to improve population health and wellbeing. This provides an evidence base to influence change across the following four MOIC strategic themes:

1. Focus on the needs of patients and the NI population.
2. Accelerate the adoption of innovation into practice to improve clinical outcomes and efficiency.
3. Build a culture of partnership and collaboration.
4. Make a meaningful contribution to the NI economy.

## Development

MOIC was established as a regional centre of expertise in NI. It is a dedicated space to develop a systematic approach to finding, testing and scaling up service and technology solutions for the Health and Social Care (HSC) service. The HSC is the term used for the National Health Service (NHS) in NI.

MOIC is uniquely positioned. NI remains in the European Union (EU) single market for goods, in the EU Customs Territory and in full regulatory alignment with the EU. As a health service organisation, MOIC facilitates access to clinicians and patients across the HSC.

### MOIC team

The Core Team is the Director, Deputy Director and Lead and a number of Senior Research and Innovation Programme Managers (educated to PhD level), Communications Manager and Secretary. The MOIC Team includes clinical pharmacy expertise, contributing to project design and delivery. This is complemented by multidisciplinary clinical expertise from across the HSC and patient representation. Two members of the MOIC Team are Health and Wellbeing Champions.

### Funding

Funding is secured from three main sources:

1. Core funding: Recurrent core funding is provided by the DH. MOIC agrees an annual business plan with the DH to undertake projects for the HSC in each financial year. The plan is informed by DH policy priorities (themes 1–3) and projects which have the potential to generate income or resources (theme 4). Additional core funding is received from the HSC Research and Development (R&D) division as part of the infrastructure spend for R&D in NI.
2. Grant acquisition: Collaborating with a range of partners and networks, MOIC applies for competitive funding schemes, including large research grants.
3. Commercial income: Working with commercial companies on projects aligned to the key themes, MOIC generates commercial income using a range of models including fee for project and revenue share. MOIC is also a knowledge provider for Invest NI. Under this arrangement, companies can apply for innovation vouchers to buy MOIC time and expertise.

### Governance

Hosted in the Northern Health and Social Care Trust (NHSCT), a major HSC Trust in NI, MOIC has a robust governance structure and oversight Programme Board, responsible for overseeing the work and strategic direction. The Programme Board, chaired by the Trust’s Accountable Officer, includes membership from across DH, healthcare, academia, industry and patient representation. Regular reports on activity and projects are provided to the Programme Board to monitor progress against the agreed business plan and to review projects undertaken via grant acquisition. For commercial projects, a bespoke project adoption process has been developed, incorporating project adoption documentation and a Project Adoption Committee. This process ensures that only projects which meet MOIC key themes are adopted. These measures ensure compliance with Regional Research and Information Governance requirements**.**

### Communications and dissemination

Communications and dissemination strategies have been designed to ensure MOIC and its work, projects, outcomes and reports are promoted extensively across a variety of media.

## Implementation

MOIC secures evidence to inform Departmental Policy and pathway redesign.

### Collaborations and partnerships

MOIC establishes collaborations, partnerships and agreements with academia, industry, healthcare and representative organisations, locally, across Ireland, Europe and beyond. These are selected based on common objectives and synergies with MOIC themes. Working collaboratively, MOIC participates in projects and shares knowledge, supporting the scale and spread of innovative solutions and best practice. Having an international reach, MOIC is also well-positioned to advise on knowledge transfer and facilitate introductions to new markets.

### Projects

In addition to local projects agreed in the annual DH business plan, MOIC works with an extensive network of partners to undertake research and service development projects. Projects range from large European grants to supporting PhD research. Arrangements are dictated by various factors, for example, the nature of MOIC involvement and relevant project requirements including those stipulated by funders. Projects span pathway redesign, clinical pharmacy, multiple long term conditions, workforce, environmental impact and sustainability, pharmacogenomics, artificial intelligence and include developing and testing integrated technology solutions and companion diagnostics.

### Industry engagement

MOIC works commercially with companies, from Small and Medium-Sized Enterprises (SMEs) to multinationals. This includes the Pharmaceutical Industry and their representative association, the Association of the British Pharmaceutical Industry (ABPI). MOIC also manages the HSC Industry Partnership (HSCIP) on behalf of NI, which aims to ensure joint working between the Pharmaceutical Industry and the HSC to deliver ‘Triple Win’ benefits for patients, the Health Service and the economy. HSCIP is a portal for collaborative proposals to work together to achieve rapid and consistent patient access to innovation, more effective use of HSC resources, increased cross-sector research collaboration and a step change in the pace and consistency of adoption of evidence-based innovative medicines and technologies.

### Hosting

A formal hosting programme has been established. Interested parties from across Europe and globally can visit MOIC, learning about its work, clinical pharmacy and related areas including antimicrobial stewardship.

### Test bed

MOIC has developed a test bed within the HSC. This facility specialises in the handling of products at a late stage in their development and almost ready to market, and offers access to HSC clinicians and patients. Partnering with the commercial sector, academia and patient representatives, MOIC combines pharmaceutical and research and development skills with technology and business acumen to improve outcomes for patients. MOIC also assists with proof of concept and signposting to appropriate individuals/entities.

## Evaluation

MOIC has demonstrated considerable success and sustainability.

### Collaborations and partnerships

**Fig. 1:**
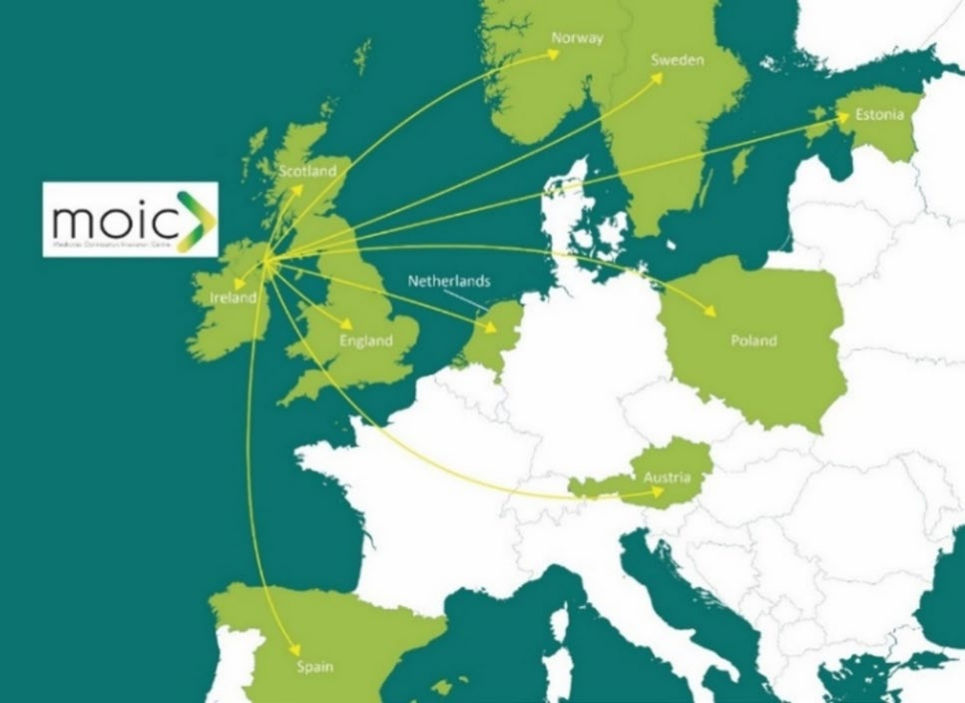
The Medicines Optimisation Innovation Centre (MOIC) European collaborations and partnerships

MOIC has established over 25 cross-sector agreements, Memoranda of Understanding and partnerships across Europe and Internationally (Fig. 1). Examples include working with the Polish Society of Clinical Pharmacy to develop clinical pharmacy; Commonwealth Pharmacists’ Association and Bugando Medical Centre, Tanzania, on antimicrobial stewardship and the development of clinical pharmacy; Cluster Saude de Galicia, Spain, on pharmacogenomics; Health Innovation Hub Ireland on clinical pharmacy and innovative technologies [12].

MOIC has also established the UK and Ireland Antimicrobial Stewardship (AMS) Collaboration, an informal network of participants with an interest in AMS and co-hosted the first All-Ireland Conference on AMS with colleagues from the Collaborate Analyse Research Audit (CARA) network.

Partnerships focusing on the Environment and Sustainability have been established across Europe, including Uppsala University, incorporating the Swedish Knowledge Centre on Pharmaceuticals in the Environment, and the One Health Breakthrough Partnership. Funding opportunities are being explored.

### Scale and spread

MOIC has informed and supported the scale and spread of best practice in clinical pharmacy and subsequent evaluation in Poland, Austria, Norway and Estonia, co-hosting its first conference in Europe focusing on clinical pharmacy in Poland in 2019 [12–17]. The NI Integrated Medicines Management model has been highlighted as an example of best practice in Central and Eastern Europe [18–20]. MOIC Co-chairs the Medicines Optimisation Thematic Working Group of the Reference Site Collaborative Network (RSCN) and NI has been recognised as a 4-star RSCN Active and Healthy Ageing Reference Site. The NI Chief Pharmaceutical Officer and MOIC have been invited to share work at the European Society of Clinical Pharmacy (ESCP) Symposium in Krakow, focusing on ‘Implementing and scaling sustainable clinical pharmacy’ [21]. ESCP advances quality and innovation in clinical pharmacy, promoting, supporting, implementing and advancing education, practice and research in clinical pharmacy to optimise outcomes for patients and society [22–32]. The work of MOIC closely aligns with ESCP aims.

### Projects

#### (i) European funded projects

MOIC has partnered in three large EU funded projects:

- SHAPES (Smart and Healthy Ageing through People Engaging in Supportive Systems): a Horizon 2020 funded, 36 partner consortium which aimed to enable older people to live healthier lives at home and build, pilot and deploy a large-scale EU-standardised open platform [33–35].
- iSIMPATHY (implementing Stimulating Innovation in the Management of Polypharmacy and Adherence Through the Years): an Interreg VA project, focussed on ensuring the best and most sustainable use of medicines by training pharmacists and other healthcare professionals to deliver structured medicine reviews and embedding a shared approach to managing multiple medicines [36, 37].
- SIMPATHY: This Horizon 2020 funded project mapped polypharmacy management and identified best practices from across the EU, provided and promoted tools and resources for different local–regional contexts to change the management of polypharmacy to increase adherence in older adults [38].

MOIC is partnering in a CwPAMS 2.0 project with colleagues from the UK and Tanzania supporting antimicrobial stewardship and clinical pharmacy. Further grant applications have been submitted across a number of areas including pharmacogenomics, multimorbidity, treatment persistency and antimicrobial stewardship. MOIC is also participating in the ENABLE COST Action focusing on technologies to support medicines adherence [39].

#### (ii) Health and social care

MOIC has led the evaluation of an array of cross-sector projects. Post discharge telephone follow up by clinical pharmacists is now routine practice in NHSCT and Consultant Pharmacists for Older People input in Nursing Homes and Intermediate Care settings has been scaled and spread across NI [40–45]. Others include a Medicines Optimisation Outpatient clinic, GP Practice-based Pharmacist case management, Pharmacy First Service in Community Pharmacy using rapid diagnostics in the management of sore throats, new models of prescribing by non-medical prescribers and the development of General Practice-based pharmacists and consultant pharmacists [46–49]. In addition to supporting the work of the NI Genomics Partnership and antimicrobial resistance initiatives, MOIC also supports the implementation of the ‘Transforming Medication Safety in Northern Ireland’ Action Plan aligned to the WHO Global Patient Safety Challenge ‘Medication Without Harm’ and the Digital Innovation Steering Group [1, 50–59].

#### (iii) PhD

MOIC co-supervises and mentors PhD students across Europe, including Poland (University of Wroclaw; Medicines Optimisation pilot), Austria (University of Ulster; Antimicrobial Stewardship and Clinical Pharmacy), Norway (University of Oslo; Transitions in Care and Multiple Long Term Conditions) and Estonia (University of Tartu; Medicine Use Reviews) [13, 14, 16, 17].

#### (iv) Quality improvement

MOIC has co-developed a novel Change Package to support the WHO Global Patient Safety Challenge ‘Medication Without Harm’ [1]. This work has been presented internationally and combines medicines optimisation approaches with quality improvement methodology. Discussions are progressing with the Institute for Healthcare Improvement and interested parties in Europe.

### Industry engagement

MOIC has completed several projects with the pharmaceutical industry. Under the HSCIP, a project is underway in cardiovascular, focusing on priority pathways. The HSCIP has generated widespread interest and work to disseminate the model is underway. Under a revenue share agreement, MOIC worked with a healthcare company in Ireland to develop smart procedure packs for blood culture, lumbar puncture, peripheral lines and HIC/PICC lines. Evaluation of the blood culture packs demonstrated a reduction in false positives, significant benefits for patients and reduced healthcare resource utilisation. The packs are now commercially available [60]. With a commercial company, MOIC co-developed and tested a solution to tag and track healthcare assets in real time in various settings. Working with a spin out company from Queen’s University Belfast, MOIC trialled a novel adherence technology to support adherence in people with respiratory disease.

### Hosting

MOIC has hosted numerous high level representatives and pharmacists from across Europe and globally.

Visitors such as Ministry of Health representatives or senior managers generally stay for a few days. Pharmacists may choose to stay for periods of up to 3 months, complete projects and take the learning back to change practice in their setting. Formal hosting arrangements are in place with organisations such as the Spanish Society of Hospital Pharmacists. MOIC is also a Statement Implementation Learning Collaborative Centre (SILCC) for the European Association of Hospital Pharmacy. This programme allows hospital pharmacists to visit SILCC hospitals to learn about pharmacy linked to the European Statements of Hospital Pharmacy.

### Covid-19

MOIC was agile during the Covid-19 pandemic. Leading work on Personal Protective Equipment procurement and informing the modelling of critical care drugs, MOIC supported the NI Covid-19 response [61, 62]. The Team also participated in the COMET European research study to establish if certain medications affect clinical outcomes in those with Covid-19 [63–65]. MOIC was commissioned by DH to support the coordination of evidence and drafting of reviews detailing the role of pharmacy in the Covid-19 response.

### Income generation

MOIC has demonstrated financial stability and sustainability and has generated income from grant acquisition and the commercialisation of solutions, contributing to the wider NI economy.

### Communication and dissemination

Disseminating extensively, including 64 journal publications, 21 reports, 121 oral and 72 poster presentations, and leveraging online, social media, electronic and print formats such as newsletters and brochures, MOIC has maximised reach. This has enabled extensive knowledge sharing and has facilitated further networks and collaborations.

### Health and wellbeing

Health, wellbeing and inclusion have been prioritised and embedded in MOIC. An Action Plan has been developed and MOIC is currently undergoing a wellbeing workplace accreditation process.

## Discussion

MOIC has achieved substantial success across all four MOIC themes and has demonstrated clear impact through driving innovation in medicines use. By optimising medicines use through practice and research to achieve person-centred and public health goals, MOIC supports innovation in Clinical Pharmacy [66].

MOIC is unique as an innovation centre focussed on medicines and associated technologies. The key facilitators for MOIC include the DH’s strategic direction and support for its work and the link to informing policy to improve patient care [67]. The person-centred approach and routine collaboration with stakeholders across academia, industry, healthcare, policy makers, patients and representative organisations are also key to MOIC’s success. Importantly, the centre is instrumental in the scale and spread of good practice which is often lacking for many small scale pilot innovations in clinical pharmacy [21]. The extensive international engagement, partnerships and reach, mean that MOIC is ideally positioned to scale and spread innovative solutions across Europe and beyond. MOIC’s HSCIP is novel and sets out a mechanism to work collaboratively with Industry with appropriate governance. This model has substantively addressed previous concerns regarding working in partnership with industry. MOICs agility and expertise was key in supporting the NI Covid-19 response.

The UK exit from the EU and uncertainty about the UK association to Horizon Europe were barriers for MOIC. The UK has now associated and MOIC can continue to participate in European funding calls. The NI Protocol has ensured that MOIC is uniquely positioned as NI remains in the EU single market for goods, in the EU Customs Territory and in full regulatory alignment with the EU. MOIC has worked diligently to communicate this association and MOIC’s unique position to colleagues across Europe. Another barrier has been the ongoing workforce and service pressures within healthcare, impacting on the availability of clinical staff to participate in projects and on hosting. To manage this, clinical expertise within MOIC is utilised and work is underway by the DH to develop the pharmacy workforce following publication of the Pharmacist Workforce Survey [68]. The hosting programme is currently paused due to ongoing service pressures within HSC, however discussions are continuing to progress the reintroduction of this popular programme.

The income generated by MOIC and its financial stability have further supported the sustainability of the centre. Not only is MOIC important for the DH, interest has also been expressed by the Department for the Economy, particularly relating to European and International engagement and discussions are underway to develop this link.

Given the unprecedented challenges faced by healthcare systems and the need for novel approaches, MOIC has generated widespread interest across Europe and beyond. Building on key successes, MOIC will continue to extend and strengthen international collaborations and partner in funding opportunities, including exploring the potential to establish a network of MOICs. MOIC is committed to improving patient care going forward through harnessing capability and innovation in pharmacogenomics, artificial intelligence, rapid diagnostics, enabling technologies, AMS and the environmental impact of medicines and sustainability.

## Conclusion

MOIC has demonstrated considerable success and sustainability, driving innovation and best practice in medicines use and associated technologies. Achieving extensive reach and recognition, MOIC is relevant and applicable to health systems and economies globally.

## References

[1] Donaldson L, Kelley E, Dhingra-Kumar N, et al. Medication Without Harm: WHO’s Third Global Patient Safety Challenge. Lancet. 2017;389(10080):1680–1. 28463129 10.1016/S0140-6736(17)31047-4

[2] Naseralallah L, Stewart D, Price M, et al. Prevalence, contributing factors, and interventions to reduce medication errors in outpatient and ambulatory settings: a systematic review. Int J Clin Pharm. 2023;45(6):1359–77. 37682400 10.1007/s11096-023-01626-5 PMC10682158

[3] World Health Organization. Global burden of preventable medication-related harm in health care: a systematic review. 2023. ISBN**:**978-92-4-008888-7. https://www.who.int/publications/i/item/9789240088887. Accessed 21 May 2024.

[4] The European Commission. A European One Health Action Plan against Antimicrobial Resistance (AMR). 2017. https://health.ec.europa.eu/system/files/2020-01/amr_2017_action-plan_0.pdf. Accessed 21 May 2024.

[5] Saeed D, Carter G, Parsons C. Interventions to improve medicines optimisation in frail older patients in secondary and acute care settings: a systematic review of randomised controlled trials and non-randomised studies. Int J Clin Pharm. 2022;44(1):15–26. 34800255 10.1007/s11096-021-01354-8 PMC8866367

[6] Northern Ireland Statistics and Research Agency. Family Practitioners Services General Pharmaceutical Services Annual Statistics 2022/23. 2023. https://bso.hscni.net/wp-content/uploads/2023/08/General-Pharmaceutical-Service-Statistics-for-NI-2022-23-Report-Accessible.pdf. Accessed 21 May 2024.

[7] Alejandre JC, Stevenson EM, Fady PE, et al. Eco-directed and Sustainable Prescribing of Pharmaceuticals in the United Kingdom: Policy Brief. 2023. https://bsac.org.uk/wp-content/uploads/2023/07/Fina-Digital_Policy-Brief-on-EDSP_18Jul23.pdf. Accessed 21 May 2024.

[8] Nazar Z, Naseralallah LM, Stewart D, et al. Application of behavioural theories, models, and frameworks in pharmacy practice research based on published evidence: a scoping review. Int J Clin Pharm. 2024;46:559–73. 38175323 10.1007/s11096-023-01674-x PMC11133055

[9] Silva L, Pacheco T, Araújo E, et al. Unveiling the future: precision pharmacovigilance in the era of personalized medicine. Int J Clin Pharm. 2024;46(3):755–60. 38416349 10.1007/s11096-024-01709-x PMC11133017

[10] Weidmann AE. Artificial intelligence in academic writing and clinical pharmacy education: consequences and opportunities. Int J Clin Pharm. 2024;46(3):751–4. 38472596 10.1007/s11096-024-01705-1 PMC11133206

[11] Department of Health, Social Services and Public Safety. Northern Ireland Medicines Optimisation Quality Framework. 2016. https://www.health-ni.gov.uk/sites/default/files/consultations/dhssps/medicines-optimisation-quality-framework.pdf. Accessed 21 May 2024.

[12] Urbańczyk K, Wnęk P, Roleder T, et al. Optimized and cost-effective pharmacotherapy of vascular surgery patients: evaluation of clinical pharmacy service. Ital J Vasc Endovasc Surg. 2022;29(2):74–9.

[13] Guntschnig S, Burnett K, Courtenay A, et al. Initial observations on the implementation of a clinical pharmacy service in a rural hospital in Austria. Hospital Pharmacy Europe. 2021;99:10–15. https://hospitalpharmacyeurope.com/reviews-research/initial-observations-on-the-implementation-of-a-clinical-pharmacy-service-in-a-rural-hospital-in-austria/. Accessed 16 May 2024.

[14] Guntschnig S, Courtenay A, Abuelhana A, et al. Clinical pharmacy interventions in an Austrian hospital: a report highlights the need for the implementation of clinical pharmacy services. Eur J Hosp Pharm. 2023. 10.1136/ejhpharm-2023-003840. 37748843 10.1136/ejhpharm-2023-003840

[15] Andersen A, Wekre L, Sund J, et al. Evaluation of implementation of clinical pharmacy services in Central Norway. Eur J Hosp Pharm. 2014;21:125–8.

[16] Tuula A, Volmer D, Jõhvik L, et al. Factors Facilitating and Hindering Development of a Medication Use Review Service in Eastern Europe and Iran-Cross-Sectional Exploratory Study. Healthcare. 2021;9(9):1207. 34574981 10.3390/healthcare9091207 PMC8468572

[17] Tuula A, Merks P, Waszyk-Nowsaczyk M, et al. Evaluation of medication safety assessments tools for pharmacist-led medication reviews; the Eastern European pilot project. Front pharmacol. 2024;15. https://www.frontiersin.org/journals/pharmacology/articles/10.3389/fphar.2024.1348400/full. Accessed 16 May 2024. 10.3389/fphar.2024.1348400 PMC10904472 38434703

[18] Scullin C, Scott M, Hogg A, et al. An innovative approach to medicines management. J Eval Clin Pract. 2007;13(5):781–8. 17824872 10.1111/j.1365-2753.2006.00753.x

[19] Scullin C, Hogg A, Luo R, et al. Integrated medicines management – can routine implementation improve quality? J Eval Clin Pract. 2012;18:807–15. 21504517 10.1111/j.1365-2753.2011.01682.x

[20] Urbańczyk K, Guntschnig S, Antoniadis V, et al. Recommendations for wider adoption of clinical pharmacy in Central and Eastern Europe in order to optimise pharmacotherapy and improve patient outcomes. Front Pharmacol. 2023;14. https://www.frontiersin.org/journals/pharmacology/articles/10.3389/fphar.2023.1244151/full. Accessed 16 May 2024. 10.3389/fphar.2023.1244151 PMC10433912 37601045

[21] Scott M, Urbańczyk K, Stewart D. European Society of Clinical Pharmacy: “Implementing and scaling sustainable clinical pharmacy.” Int J Clin Pharm. 2024;46(2):355–6. 38478210 10.1007/s11096-024-01718-w

[22] Moura L, Steurbaut S, Salvesen Blix H, et al. A cross-sectional survey to map Clinical Pharmacy Education and Practice in Europe. Int J Clin Pharm. 2022;44:118–26. 34498216 10.1007/s11096-021-01321-3

[23] Apikoglu S, Selcuk A, Ozcan V, et al. The first nationwide implementation of pharmaceutical care practices through a continuing professional development approach for community pharmacists. Int J Clin Pharm. 2022;44:1223–31. 35699862 10.1007/s11096-022-01413-8 PMC9194772

[24] Mantzourani E, Brooks O, James D, et al. Development, implementation and evaluation of the digital transformation of renal services in Wales: the journey from local to national. Int J Clin Pharm. 2023;45:4–16. 36306061 10.1007/s11096-022-01466-9 PMC9614750

[25] Cheng C, Walsh A, Jones S, et al. Development, implementation and evaluation of a seven-day clinical pharmacy service in a tertiary referral teaching hospital during surge-2 of the COVID-19 pandemic. Int J Clin Pharm. 2023;45:293–303. 36367601 10.1007/s11096-022-01475-8 PMC9650667

[26] Tait LA, Cassidy N, Jamieson D, et al. Medication supply at hospital discharge via community pharmacy: a quality improvement study. Int J Clin Pharm. 2023;45:1309–16. 37768432 10.1007/s11096-023-01635-4

[27] Howlett MM, Sutton S, McGrath EL, et al. Implementation of a national system for best practice delivery of paediatric infusions. Int J Clin Pharm. 2024;46:4–13. 37971685 10.1007/s11096-023-01652-3

[28] Stuhec M, Hahn M, Taskova I, et al. Clinical pharmacy services in mental health in Europe: a commentary paper of the European Society of Clinical Pharmacy Special Interest Group on Mental Health. Int J Clin Pharm. 2023;45:1286–92. 37755642 10.1007/s11096-023-01643-4 PMC10600282

[29] Weidmann AE, Cadogan CA, Fialová D, et al. How to write a successful grant application: guidance provided by the European Society of Clinical Pharmacy. Int J Clin Pharm. 2023;45:781–6. 36877291 10.1007/s11096-023-01543-7 PMC10250258

[30] Wirth F, Cadogan CA, Fialová D, et al. Writing a manuscript for publication in a peer-reviewed scientific journal: guidance from the European Society of Clinical Pharmacy. Int J Clin Pharm. 2024;46:548-54. 10.1007/s11096-023-01695-6 PMC10960906 38332208

[31] Paudyal V, Okuyan B, Henman MC, et al. Scope, content and quality of clinical pharmacy practice guidelines: a systematic review. Int J Clin Pharm. 2024;46:56–69. 37991663 10.1007/s11096-023-01658-x PMC10830799

[32] Fernandez-Llimos F, Desselle S, Stewart D, et al. Improving the quality of publications in and advancing the paradigms of clinical and social pharmacy practice research: the Granada Statements. Int J Clin Pharm. 2023;45:285–92. 36920737 10.1007/s11096-023-01550-8 PMC10147809

[33] Seidel K, Labor M, Lombard-Vance R, et al. Implementation of a pan-European ecosystem and an interoperable platform for Smart and Healthy Ageing in Europe: An Innovation Action research protocol. Open Res Europe. 2022;2:85. 10.12688/openreseurope.14827.1 PMC10446093 37645338

[34] Spargo M, Goodfellow N, Scullin C, et al. Shaping the Future of Digitally Enabled Health and Care. Pharmacy. 2021;9(1):17. 33445509 10.3390/pharmacy9010017 PMC7838996

[35] Spargo M, Goodfellow N, Scott M, et al. SHAPES D6.4–Medicine Control and Optimisation Pilot Activities Report. 2023. shapes2020.eu/wp-content/uploads/2023/09/857159_Deliverable_62_Medicine-Control-and-Optimisation-Pilot-Activities-Report.pdf. Accessed 16 May 2024.

[36] The iSIMPATHY Consortium. iSIMPATHY Evaluation Report. 2023. https://www.isimpathy.eu/uploads/iSIMPATHY_Evaluation_report_ver8_online.pdf. Accessed 16 May 2024.

[37] Brown J, Hogg A, Scullin C, et al. 7-Steps medication reviews: analysis of medicine changes in acute medical wards. Int J Pharm Pract. 2022;30(2):ii7–ii8.

[38] Mair A, Fernandez-Llimos F. SIMPATHY Consortium. Polypharmacy management programmes: the SIMPATHY Project. Eur J Hosp Pharm. 2017;24(1):5–6. 31156889 10.1136/ejhpharm-2016-001044 PMC6451609

[39] European Cooperation in Science and Technology. Enable Adherence. 2024. https://enableadherence.eu/. Accessed 21 May 2024.

[40] Odeh M, Scullin C, Fleming G, et al. Ensuring continuity of patient care across the healthcare interface: Telephone follow-up post-hospitalization. Br J Clin Pharmacol. 2019;85(3):616–25. 30675742 10.1111/bcp.13839 PMC6379220

[41] McKee H, Miller R, Cuthbertson J, et al. Nursing Home Outreach Clinics show an improvement in patient safety and reduction in hospital admissions in residents with chronic conditions. EJPCH. 2016;4(4):650–5.

[42] Miller R, Darcy C, Friel A, et al. Consultant pharmacist case management of older people in intermediate care: a new innovative model. Eur J Pers Cent Healthc. 2016;4(1):46–52.

[43] Miller R, Darcy C, McGeough N, et al. The Development and Refinement of a Regional Model for Medicines Optimisation in Older People in the Intermediate Care Setting. Int J Integr Care. 2017;17(5):A120.

[44] Miller R, McKee H, Friel A, et al. Developing a Regional Medicines Optimisation Model for Older People in Care Homes: Refinement and Reproducibility. Int J Integr Care. 2017;17(5):A121.

[45] Miller R, McClean M, Friel A. An Independent Service User Evaluation of a Consultant Pharmacist Led Medicines Optimisation in Older People’s Project. Int J Integr Care. 2017;17(5):A122.

[46] Odeh M, Scullin C, Hogg A, et al. A novel approach to medicines optimisation post-discharge from hospital: pharmacist-led medicines optimisation clinic. Int J Clin Pharm. 2020;42(4):1036–49. 32524511 10.1007/s11096-020-01059-4 PMC7476989

[47] Syafhan N, Al Azzam S, Williams SD, et al. General practitioner practice-based pharmacist input to medicines optimisation in the UK: pragmatic, multicenter, randomised controlled trial. J Pharm Policy Pract. 2021;14(1):4. 33397509 10.1186/s40545-020-00279-3 PMC7784025

[48] O’Neill K, Fleming G, Scott MG, et al. C-Reactive Protein Point of Care Testing in Community Pharmacy: Observational study of a Northern Ireland Pilot. Pharm Pract. 2022;20(4):2711. 10.18549/PharmPract.2022.4.2711 PMC9891790 36793914

[49] Gormley C, Spargo M, Fleming G, et al. Medicines Optimisation for Respiratory Patients The Establishment of a New Consultant Respiratory Pharmacist Role in Northern Ireland. Pharmacy. 2021;9(4):177. 34842802 10.3390/pharmacy9040177 PMC8629016

[50] Conlon-Bingham G, Aldeyab M, Kearney M, et al. Reduction in the incidence of hospital-acquired MRSA following the introduction of a Chlorine Dioxide 275 ppm based disinfecting agent in a district general hospital. Eur J Hosp Pharm. 2016;23(1):28–32. 31156810 10.1136/ejhpharm-2014-000608 PMC6451552

[51] Al-Taani G, Scott M, Farren D, et al. Longitudinal point prevalence survey of antibacterial use in Northern Ireland using the European Surveillance of Antimicrobial Consumption (ESAC) PPS and Global-PPS tool. Epidemiol & Infection. 2018;25:1–6. 10.1017/S095026881800095X PMC9184955 29690946

[52] Khdour M, Hallak H, Aldeyab M, et al. Impact of Antimicrobial Stewardship program on Hospitalized Patients at the Intensive Care Unit: A prospective audit and feedback study. Br J Clin Pharmacol. 2018;84:708–15. 10.1111/bcp.13486 PMC5867097 29236303

[53] Elhajji FD, Al-Taani GM, Anani L, et al. Comparative point prevalence survey of antimicrobial consumption between a hospital in Northern Ireland and a hospital in Jordan. BMC Health Serv Res. 2018;18(1):849. 30419895 10.1186/s12913-018-3656-y PMC6233602

[54] Conlon-Bingham G, Aldeyab M, Scott M, et al. Effects of Antibiotic Cycling Policy on Incidence of Healthcare-Associated MRSA and Clostridioides difficile Infection in Secondary Healthcare Settings. Emerg Infect Dis. 2019;25(1):52–62. 30561306 10.3201/eid2501.180111 PMC6302607

[55] López-Lozano J, Lawes T, Nebot C, et al. A nonlinear time-series analysis approach to identify thresholds in associations between population antibiotic use and rates of resistance. Nat Microbiol. 2019;4:1160–72. 30962570 10.1038/s41564-019-0410-0

[56] Alnajjar M, Aldeyab M, Scott M, et al. Influence of primary care antibiotic prescribing on incidence rates of multidrug-resistant Gram-negative bacteria in hospitalised patients. Infection. 2019;47(5):781–91. 31065996 10.1007/s15010-019-01305-6

[57] Jirjees F, Al-Obaidi H, Sartaj M, et al. Antibiotic use and resistance in hospitals: time-series analysis strategy for determining and prioritising interventions. Hospital Pharmacy Europe. 2020;95:13–19. https://hospitalpharmacyeurope.com/news/reviews-research/antibiotic-use-and-resistance-in-hospitals-time-series-analysis-strategy-for-determining-and-prioritising-interventions/. Accessed 21 May 2024.

[58] Transforming medication safety in Northern Ireland. Department of Health. 2018. https://www.health-ni.gov.uk/sites/default/files/publications/health/Transforming-medication-safety-in-Northern-Ireland_1.pdf. Accessed 23 May 2024.

[59] Digital strategy Health and Social Care Northern Ireland 2022–2030. Department of Health. 2022. https://www.health-ni.gov.uk/digitalstrategy. Accessed 23 May 2024.

[60] Alahmadi Y, McElnay J, Kearney M, et al. Tackling the problem of blood culture contamination in the intensive care unit using an educational intervention. Epidemiol Infect. 2015;143(9):1964–71. 25387485 10.1017/S0950268814003008 PMC9507266

[61] Burnett K, Martin S, Goudy C, et al. Ensuring the quality and quantity of personal protective equipment (PPE) by enhancing the procurement process in Northern Ireland during the coronavirus disease 2019 pandemic: challenges in the procurement process for PPE in NI. J Patient Saf Risk Manag. 2021;21(7):42–9. 10.1177/25160435211057385 PMC8926917 35317420

[62] Hogg A, Huey R, Scott M, Ferguson A. Informing Critical Care Drug Requirements in Response to the COVID-19 Pandemic. Eur J Hosp Pharm. 2020;27(5):263–6. 32661105 10.1136/ejhpharm-2020-002368 PMC7371569

[63] Sablerolles R, Hogenhuis F, Lafeber M, et al. COvid MEdicaTion (COMET) study: protocol for a cohort study. Eur J Hosp Pharm. 2020;27(4):191–3. 32587077 10.1136/ejhpharm-2020-002329 PMC7335622

[64] Sablerolles R, Hogenhuis F, Lafeber M, et al. No association between use of ACE inhibitors or angiotensin II receptor blockers prior to hospital admission and clinical course of COVID-19 in the COvid MEdicaTion (COMET) study. Br J Clin Pharmacol. 2021;87(8):3301–9. 33507556 10.1111/bcp.14751 PMC8014637

[65] Sablerolles R, Lafeber M, van Kempen J, et al. Association between Clinical Frailty Scale score and hospital mortality in adult patients with COVID-19 (COMET): an international, multicentre, retrospective, observational cohort study. Lancet Healthy Longev. 2021;2(3):e163–70. 33655235 10.1016/S2666-7568(21)00006-4 PMC7906710

[66] Dreischulte T, van den Bemt B, Steurbaut S, et al. European Society of Clinical Pharmacy definition of the term clinical pharmacy and its relationship to pharmaceutical care: a position paper. Int J Clin Pharm. 2022;44:837–42. 35668277 10.1007/s11096-022-01422-7 PMC9393137

[67] Al Bulushi S, McIntosh T, Grant A, et al. Implementation frameworks for polypharmacy management within healthcare organisations: a scoping review. Int J Clin Pharm. 2023;45(2):342–54. 36719590 10.1007/s11096-023-01534-8 PMC10147734

[68] Department of Health. The Northern Ireland Pharmacist Workforce Survey 2022–Results. 2022. https://www.health-ni.gov.uk/publications/pharmacist-workforce-survey. Accessed 23 May 2024.

